# A Highly Stretchable, Tough, Fast Self-Healing Hydrogel Based on Peptide–Metal Ion Coordination

**DOI:** 10.3390/biomimetics4020036

**Published:** 2019-05-10

**Authors:** Liang Zeng, Mingming Song, Jie Gu, Zhengyu Xu, Bin Xue, Ying Li, Yi Cao

**Affiliations:** 1 Collaborative Innovation Center of Atmospheric Environment and Equipment Technology, Jiangsu Key Laboratory of Atmospheric Environment Monitoring and Pollution Control, School of Environmental Science and Engineering, Nanjing University of Information Science & Technology, Nanjing 210044, China; 20161244375@nuist.edu.cn; 2 Collaborative Innovation Center of Advanced Microstructures, National Laboratory of Solid State Microstructure, Key Laboratory of Intelligent Optical Sensing and Manipulation, Ministry of Education, Department of Physics, Nanjing University, Nanjing 210093, China; 151120102@smail.nju.edu.cn (M.S.); 131242017@smail.nju.edu.cn (J.G.); 151242051@smail.nju.edu.cn (Z.X.)

**Keywords:** self-healing, hydrogel, stretch-ability, metal ion coordination

## Abstract

Metal coordination bonds are widely used as the dynamic cross-linkers to construct self-healing hydrogels. However, it remains challenging to independently improve the toughness of metal coordinated hydrogels without affecting the stretchability and self-healing properties, as all these features are directly correlated with the dynamic properties of the same metal coordination bonds. In this work, using histidine–Zn^2+^ binding as an example, we show that the coordination number (the number of binding sites in each cross-linking ligand) is an important parameter for the mechanical strength of the hydrogels. By increasing the coordination number of the binding site, the mechanical strength of the hydrogels can be greatly improved without sacrificing the stretchability and self-healing properties. By adjusting the peptide and Zn^2+^ concentrations, the hydrogels can achieve a set of demanding mechanical features, including the Young’s modulus of 7–123 kPa, fracture strain of 434–781%, toughness of 630–1350 kJ m^−3^, and self-healing time of ~1 h. We anticipate the engineered hydrogels can find broad applications in a variety of biomedical fields. Moreover, the concept of improving the mechanical strength of metal coordinated hydrogels by tuning the coordination number may inspire the design of other dynamically cross-linked hydrogels with further improved mechanical performance.

## 1. Introduction

Self-healing hydrogels have received considerable attention in recent years due to their diverse biomedical applications, such as wound dressing, soft tissue medical adhesives, injectable drug carriers, and self-supporting 3D printing inks [1,2,3,4,5,6,7,8]. Generally, self-healing hydrogels can use either dynamic covalent or noncovalent cross-linkers as the healing motifs. Dynamic covalent cross-linkers include boronate ester bonds [9,10,11,12,13,14], imine bonds [15,16], acylhydrazone bonds [17,18], and oxime bonds [19], etc. These bonds are chemically labile under physiological conditions. Despite that the hydrogels cross-linked by dynamic covalent cross-linkers possess high mechanical stability, they self-heal slowly, as typical dynamic covalent bonds show very slow exchange dynamics. On the other hand, physical cross-linkers, including hydrophobic effects [20,21,22,23], hydrogen bonding [24,25,26,27,28,29], host–guest interactions [30,31,32], ionic bonding [33], metal coordination [34,35,36,37,38,39,40,41,42,43,44,45,46], and specific protein–protein interactions [47,48,49], are highly dynamic, rendering the hydrogels with great self-healing properties. However, they are typically too dynamic to give rise to mechanically tough hydrogels, because mechanical strength of hydrogels is also directly correlated with the cross-linker dynamics [34,50,51,52]. Therefore, in most self-healing materials, the toughness and the healing rates are compromised. It remains challenging to engineer mechanically tough yet highly stretchable and fast healable hydrogels.

Recently Holten-Andersen et al. experimentally [53], and Hartmann et al. theoretically [54], showed that the coordination number, which is the number of ligands involved in the cross-linking junction, is critical for the mechanical properties of the resulting hydrogels. By increasing the number of cross-linking sites using Fe_3_O_4_ nanoparticles instead of Fe^3+^ ions with catechol, the hydrogels became much tougher yet remained reversible [53]. Increasing the coordination number has a beneficial effect on the mechanical strength of the hydrogels due to the cooperative rupture of these bonds upon loading [54].

Inspired by these studies, here we report on the construction of highly stretchable, tough and fast self-healing hydrogels cross-linked by peptide–metal ion coordination sites with high coordination number (the number of binding site in each cross-linking ligand). We show that comparing to the hydrogels cross-linked by single ligand–metal ion bonds, the peptide–metal ion coordinated hydrogels show much higher Young’s modulus and break strength as well as similar break strain and self-healing rate. We anticipate that these hydrogels can find broad biomedical applications in tissue engineering, wound addressing and 3D bioprinting.

## 2. Materials and Methods

### 2.1. Materials

Acrylamide was purchased from Sigma-Aldrich (Shanghai, China). Zinc chloride (ZnCl_2_), potassium chloride (KCl), ammonium persulfate (APS), hydrochloric acid (HCl), and tris(hydroxymethyl)methyl aminomethane (Tris base) were purchased from Sinopharm Chemical Regent Co., Ltd. (Beijing, China). The designed peptides were purchased from GL Biochem (Shanghai) Ltd. (Shanghai, China). All regents were used without further purification.

### 2.2. Preparation of the Gly-Gly-His (GGH) and Gly-His-His-Pro-His (GHHPH) Hydrogels

In a typical preparation of hydrogels, peptides and acrylamide were dissolved in Milli-Q water to the concentrations of 100 mg mL^−1^ and 50 mg mL^−1^, respectively. The solution was degassed three times for 15 min by ultrasound under argon atmosphere. Ammonium persulfate was added as a photoinitiator for polymerization. The solutions were filled into the molds and polymerization was undertaken under ultraviolet light (285 nm) irradiation for 20 h. Then the hydrogels were immersed in 1 M Tris buffer (pH = 7.60, containing 300 mM of KCl and different molars of ZnCl_2_) for 24 h to form the coordinate bonds. The solid content (W_s_) of the hydrogels was calculated as $W_{s}=\frac{W_{d}}{W_{0}}$, where *W*_d_ is the weight of the lyophilized hydrogel and *W*_0_ corresponds to the weight of the corresponding wet hydrogel. The molecular weights of the polymers were characterized using gel permeation chromatography (GPC) (LC-20A, Shimadzu, Japan) in THF with tandem Shodex KF-803 and KF-805 columns. The molecular weights were calibrated using the polystyrene molecular weight standard (ZZStandard, Shanghai, China). Then the degree of polymerization was calculated based on the molecular weights of the polymers and the molar ratio of peptides and acrylamide in the hydrogels.

### 2.3. Rheological Measurements

The rheological measurements were carried out on a standard rheometer (Thermo Scientific Haake RheoStress 6000, Thermo, Karlsruhe, Germany). The hydrogels were carefully placed on the rheometer plate and allowed to equilibrate for ~30 min prior to the measurements. The rheology experiments were then carried out either using a strain sweep mode with a strain amplitude range of 0.01% to 100% at a fixed frequency of 6.28 rad s^−1^ or using a frequency sweep mode with a frequency range of 0.01 rad s^−1^ to 100 rad s^−1^ at a fixed strain of 0.1% (geometry: 1°/20 mm of cone and plate; gap: 0.37 mm; temperature: 20 °C). For the recovery experiments, the G’ and G’’ were measured at a strain of 0.1% and a frequency of 6.28 rad s^−1^. Then strain was set to an amplitude of 1000% to destroy the hydrogels for 60 s and switched back to an amplitude of 0.1% to monitor the recovery of the mechanical properties for 300 s.

### 2.4. Tensile and Compressive Test

The tensile stress–strain measurements were performed on a tensile–compressive tester (Instron-5944 with a 10 N sensor, Instron, Boston, MA, USA) in air at room temperature. In the tension crack test, the strain rate of stretching was maintained at different percentages of the original length of the samples per minute and the original length of the hydrogel samples in the uniaxial tension ranged from 7.5 mm to 10.0 mm. The tensile toughness was calculated from the area below the stress–strain curve until fracture. The equation used in the calculation was as below; $E_{f}=\int_{x_{0}}^{x_{f}}\sigma \left(x\right)dx$ in which $x_{0}$ corresponds to the starting point of the tension, corresponds to the fracture point of the tension and *σ* corresponds to the stress during the tension. [55,56,57] The Young’s moduli, E, were measured by fitting the linear region of the stress–strain curve (below 20% strain). The definitions of all the parameters and formulations are listed in Table S1.

### 2.5. Scanning Electron Microscopy (SEM) measurements

SEM images were obtained using a Quanta Scanning Electron Microscope (Quata 200, FEI, Thermo, Hillsboro, OR, USA) at 20 kV. The hydrogels were lyophilized prior to the measurement.

## 3. Results

### 3.1. Results and Analysis

#### 3.1.1. The Design and Preparation of the Self-Healing Hydrogels

**Figure 1:**
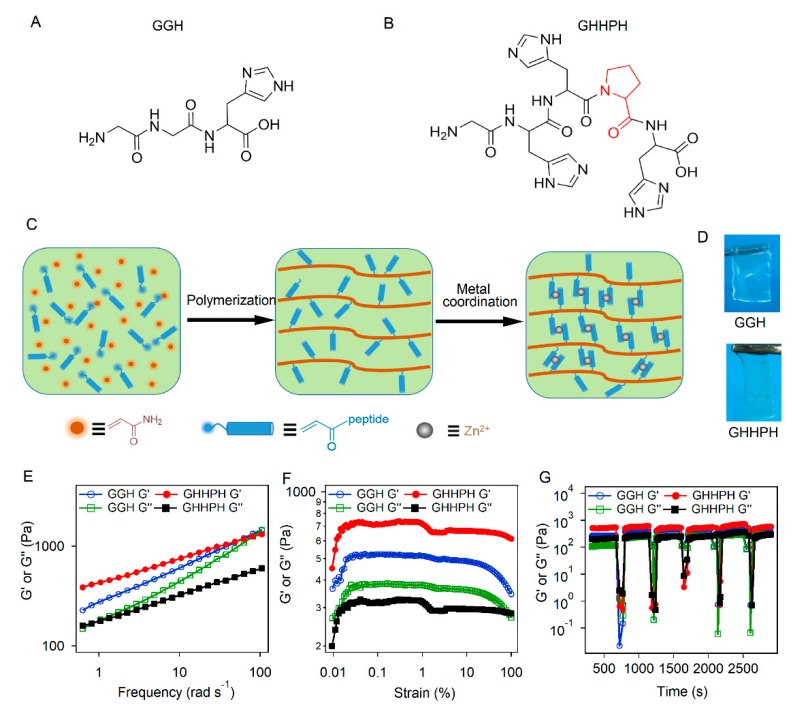
Illustrative scheme and rheological mechanical properties of the self-healing hydrogels based on the coordination interactions between peptides and Zn^2+^ ions. (**A**) Chemical structure of the GGH peptide. (**B**) Chemical structure of the GHHPH peptide. (**C**) Schematic illustration of the hydrogels based on the coordination interactions between the designed peptides and Zn^2+^ ions. (**D**) Images corresponding to GGH and GHHPH hydrogels. (**E**) G’ and G” of the hydrogels measured in a frequency sweep experiment (frequencies from 0.01 to 100 rad s^−1^, 0.1% strain) at the peptide concentration of 50 mg mL^−1^ while the concentration of acrylamide was always 25 mg mL^−1^. The molar ratios of peptides and ZnCl_2_ in GGH and GHHPH hydrogels were 15:3 and 15:9, respectively. (**F**) G’ and G” of the hydrogels measured in a strain sweep experiment (strains from 0.01 to 100%, a frequency of 6.28 rad s^−1^) at the peptide concentration of 50 mg mL^−1^, while the concentration of acrylamide was always 25 mg mL^−1^ and molar ratio of peptides and ZnCl_2_ were 15:3 and 15:9 for GGH and GHHPH hydrogels, respectively. (**G**) G’ and G” of the hydrogels measured in a destroy–recovery experiment at the peptide concentration of 50 mg mL^−1^ while the concentration of acrylamide was always 25 mg mL^−1^ and molar ratios of peptides and ZnCl_2_ were 15:3 and 15:9 for GGH and GHHPH hydrogels, respectively. The strain was set to an amplitude of 1000% for 60 s to destroy the coordination interactions and switched back to an amplitude of 0.1% to monitor the recovery of the mechanical properties for 600 s. The G’ and G’’ were measured at a frequency of 6.28 rad s^−1^ at 20 °C.

Two kinds of short peptides containing different number of metal ion binding sites (histidine) [58,59] were used as the cross-linkers in the self-healing hydrogels. The sequences of the two peptides are Gly–Gly–His (GGH) and Gly–His–His–Pro–His (GHHPH), respectively (Figure 1A,B). The histidine residues in both peptides can form coordination bonds with Zn^2+^ ions. The GGH peptide contains only a single histidine and forms a single coordination bond with Zn^2+^ ions. In contrast, GHHPH can bind with Zn^2+^ ions with a 2:1 stoichiometric ratio as previously reported [60], indicating that GHHPH forms at least two coordination bonds with Zn^2+^, exhibiting higher coordination number (the number of binding site in each cross-linking ligand) than GGH. Although metal coordination bonds can be directly conjugated to multiarmed hydrophilic polymers to form metal chelating hydrogels, the mechanical properties of those networks were relatively weak due to the limited cross-linking density [34,44]. We therefore used an aqueous free radical copolymerization approach to polymerize the hydrophilic monomer (acrylamide) and the acryloyl-terminated peptides directly into an entangled weak network of pre-gels (Figure 1C). The pre-gels were in the boundary of hydrogels and viscous solutions whose storage moduli were close to the loss moduli in a broad frequency range (Figure S1). There was no inter-chain covalent cross-linking in the pre-gels. Then, the pre-gels were immersed in Tris buffer (pH = 7.60, 100 mM Tris and 300 mM KCl) containing different concentrations of ZnCl_2_ for 48 h in order to form coordination bonds between peptides and Zn^2+^ ions (Figure 1C). The hydrogels (denoted as GGH and GHHPH hydrogels) were transparent and free-standing (Figure 1D). The microstructures of the hydrogels were studied by scanning electron microscopy (SEM). Both the two kinds of hydrogels exhibited similar microstructures of porous networks (Figure S2).

#### 3.1.2. Dynamic Mechanical Properties of the Self-Healing Hydrogels

The dynamic mechanical properties of the two kinds of hydrogels were investigated using dynamic shear rheology. As shown in Figure 1E, the G’ (storage modulus) of all hydrogels was obviously larger than G’’ (loss modulus), indicating solid rather than viscous response of the gel. The G’ increased with the increase of peptide concentrations (Figure S3). The G’ and G’’ of GHH hydrogels crossed at a frequency of ~100 rad s^−1^. This suggested that the histidine–Zn^2+^ coordination bonds in both hydrogels were highly dynamic with a characteristic lifetime shorter than 0.03 s. In comparison, GHHPH hydrogels were stiffer than GGH hydrogels at the same peptide concentrations, as evidenced by their higher storage modulus G’ (Figure 1E and Figure S3). No crossover of G’ and G’’ was observed, suggesting that the binding of GHHPH became much static. Moreover, the strain sweep experiments were also performed (Figure 1F). The yield strength values of GGH and GHHGH hydrogels were 354 Pa and 432 Pa, respectively, indicating that both the gels can undergo plastic deformation at low amplitudes (Figure 1F). Furthermore, all hydrogels, except for GGH hydrogel at the low peptide concentration of 25 mg mL^−1^, can survive up to 100% strain, suggesting that the hydrogels were mechanically quite robust (Figure S4). For comparison, the covalent hydrogels cross-linked by bis-acrylamide with the same cross-linking density was studied as the control group (Figure S5A). The G’ and G’’ of the hydrogel with covalent network were similar with those of GGH and GHHGH hydrogels.

Then the reassociation of the peptide-Zn^2+^ bonds was studied after a large strain oscillatory breakdown at 20 °C (Figure 1G and Figure S6). Both GGH and GHHPH hydrogels can be fractured at strains higher than 1000% due to the mechanical rupture of the peptide-Zn^2+^ cross-linkers. However, both the G’ and G’’ can be fully recovered in a few seconds, suggesting that these hydrogels may own self-healing properties owing to the fast reassociation of the coordination bonds. The recovery of GHHPH hydrogels was even faster, suggesting that the histidine residues in the peptide may exhibit certain cooperativity for Zn^2+^ binding.

#### 3.1.3. Tensile Mechanical Properties of the Self-Healing Hydrogels

**Figure 2:**
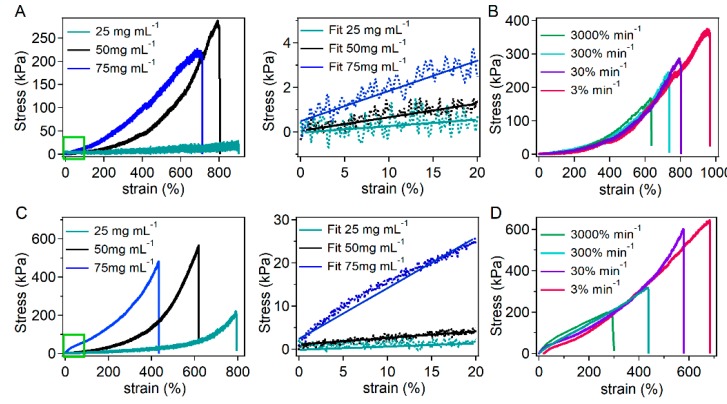
Tensile mechanical properties of GGH and GHHPH hydrogels. (**A**) Stress–strain curves (left) of GGH hydrogels and zoomed stress–strain curves (right) at the strain rate of 30% min^−1^ with different mass ratios of GGH and acrylamide (1:1, 2:1, and 3:1). The solid lines in the right indicate the linear fitting of the elastic region. The concentrations of GGH peptides were 25 mg mL^−1^, 50 mg mL^−1^, and 75 mg mL^−1^, respectively, while the concentration of acrylamide was always 25 mg mL^−1^ and the molar ratios of peptides and ZnCl_2_ were always 15:3. (**B**) Stress–strain curves of GGH hydrogels (*m*_GGH_:*m*_acrylamide_= 2:1) with different tensile strain rates (3%, 30%, 300% and 3000% per minute). (**C**) Stress–strain curves (left) of GHHPH hydrogels and zoomed stress–strain curves (right) at the strain rate of 30% min^−1^ with different mass ratios of GHH and acrylamide (1:1, 2:1, and 3:1). The solid lines in the right indicate the linear fitting of the elastic region. The concentrations of GHHPH peptides were 25 mg mL^−1^, 50 mg mL^−1^, and 75 mg mL^−1^, respectively, while the concentration of acrylamide was always 25 mg mL^−1^ and the molar ratios of peptides and ZnCl_2_ were always 15:9. (**D**) Stress–strain curves of GHHPH hydrogels (*m*_GHHPH_:*m*_acrylamide_ = 2:1) with different tensile strain rates (3%, 30%, 300% and 3000% min^−1^).

Next, the tensile mechanical properties of the hydrogels were measured by standard tensile tests. The typical tensile stress–strain curves with different mass ratios of the acryloyl-terminated peptide and acrylamide (*w/w* 1:1, 2:1 and 3:1) are shown in Figure 2A,C and the degree of polymerization, solid content, and mechanical properties were summarized in Table 1. Similar to the rheological properties, the tensile mechanical properties, including fracture stress, Young’s modulus, and toughness of GHHPH hydrogels were systematically higher than that of GGH hydrogels. The maximum toughness of GGH hydrogels was 868.07 kJ m^−3^ while that of GHHPH hydrogels reached more than 1300 kJ m^−3^. Increasing peptide concentrations led to higher stiffness and toughness of the hydrogels, suggesting that the mechanical properties can be manipulated by adjusting the density of the cross-linkers. It is worth mentioning that the solid contents of these hydrogels were different and in a range of 22.79% to 31.69% (Table 1). However, due to the different Zn^2+^ binding ability and swelling ratios of these hydrogels, the solid content was difficult to predict a priori. Nonetheless, the dramatic difference in Young’s moduli and strain limit for all these hydrogels cannot be attributed to the variation of the solid contents. The different coordination numbers of the cross-linkers played the major role. In comparison, the stretchability and toughness of the covalent hydrogels were much lower than that of GGH and GHHGH hydrogels (Figure S5B).

Furthermore, the mechanical properties of the hydrogels with different tensile strain rates were also studied in order to determine the reversibility of the cross-linkers. As shown in Figure 2B,D, the fracture strain of both kinds of hydrogels became larger as the tensile strain rate decreased, indicating that the toughness of the hydrogels increased as the strain rate increased. However, the Young’s modulus of the hydrogels almost remained unchanged with the increase of strain rates, suggesting that the change of strain rates would not affect the elasticity of the hydrogels. The independent control of the toughness and elasticity demonstrated that the cross-links based on the coordination between the designed peptides and Zn^2+^ ions were reversible, which is consistent with recent experimental studies [61,62] and theoretic work [63]. According to the theory [63], to have the strain rate independent elasticity, the strain rate timescale should be longer than the timescale of reversible dissociation/association of the cross-linkers. Therefore, the coordination bonds between GGH/GHHPH and Zn^2+^ have reversible dynamics at a timescale shorter than 2 s. Due to the limited strain rate available in our tensile test machine, which cannot go beyond 3000% min^−1^ in the experiments, the exact reversible dynamics cannot be obtained. Nonetheless, it is clear that GHHPH can still retain high dissociation/association rates with Zn^2+^ in the cross-linking site, which is the prerequisite for fast self-healing.

At last, the tensile experiments with multiple cyclic loading were also performed to investigate the dissipated energy, fatigue, and plastic deformation (Figure S7). The energy dissipation of GGH and GHHGH hydrogels reached 172.4 kJ m^−3^ and 413.0 kJ m^−3^, respectively, in the first tensile cycle, exhibiting excellent energy dissipation properties due to the absence of covalent cross-linkers. The dissipated energy and maximum stress changes are also summarized in Figure S7C,D. The dissipated energy as well as the maximum stress decreased significantly after multiple cyclic loading. However, the dissipated energy remained more than 20% after seven tensile cycles, indicating that the peptide-Zn^2+^ coordination bonds can partially reform during relaxation. The decrease of dissipated energy and maximum stress suggested the fast accumulation of plastic deformation and fatigue of the hydrogels.

#### 3.1.4. Self-Healing Properties of the Hydrogels

**Figure 3:**
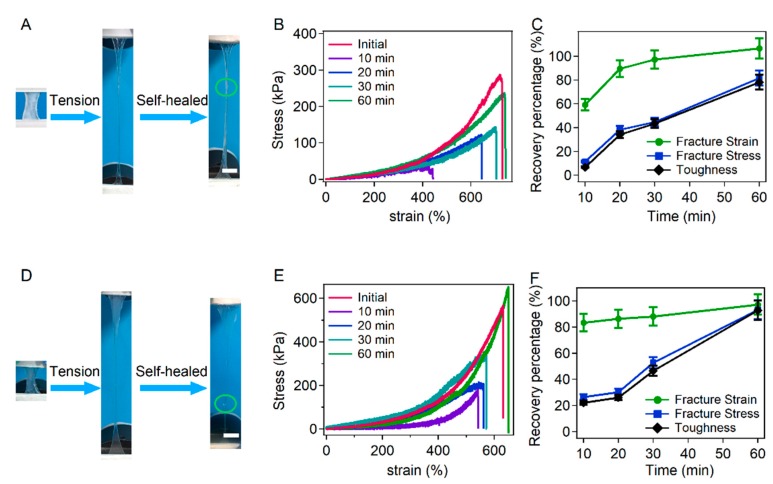
Self-healing properties of GGH and GHHPH hydrogels. (**A**) Stretching photograph of the GGH hydrogel before and after healing. The reconnected points are highlighted with a green cycle. Scale bar = 5 mm. (**B**) Stress–strain curves of GGH hydrogels at the strain rate of 30% min^−1^ healed for different times at room temperature. The concentrations of GGH peptides and acrylamide were 50 mg mL^−1^ and 25 mg mL^−1^, respectively, while the molar ratios of peptides and ZnCl_2_ were always 15:3. (**C**) Normalized recovery percentages of fracture strain, fracture stress and toughness with different healing time corresponding to (B). (**D**) Stretching photograph of the GHHPH hydrogel before and after healing. The reconnected points are highlighted with a green cycle. Scale bar = 5 mm. (**E**) Stress–strain curves of GHHPH hydrogels at the strain rate of 30% min^−1^ healed for different times at room temperature. The concentrations of GHHPH peptides and acrylamide were 50 mg mL^−1^ and 25 mg mL^−1^_,_ respectively, while the molar ratios of peptides and ZnCl_2_ were always 15:9. (**F**) Normalized recovery percentages of fracture strain, fracture stress and toughness with different healing time corresponding to (E).

Next, we tested the self-healing properties of the hydrogels. In a typical self-healing experiment, the hydrogel was cut into two pieces and put together to self-heal for different times under slight pressure provided by two parallel disk plates in Tris buffer (pH = 7.60, containing 100 mM Tris and 300 mM KCl). The representative images of hydrogels before and after healing, stress–strain curves and normalized recovery percentage of the mechanical properties are illustrated in Figure 3. Obviously, longer healing time led to higher recovery percentages. For GGH hydrogels, the fracture strain can reach the initial level after healing for 60 min and the fracture stress and toughness can reach ~80% of the initial level. For GHHPH hydrogels, the fracture strain can be fully recovered after healing for 60 min and the fracture stress and toughness can reach about 90% of the initial level. The self-healing of the GHHPH hydrogels was slightly higher than that of GGH hydrogels, probably due to higher cooperativity of the bonds between GHHPH and Zn^2+^ ions than that of GGH and Zn^2+^. These experiments confirmed that the hydrogels based on the coordination bonds between GHHPH and Zn^2+^ ions showed combined high stretchability, great mechanical strength and fast self-healing properties. In comparison, no self-healing property was observed in the covalent hydrogels (Figure S5C).

**Figure 4:**
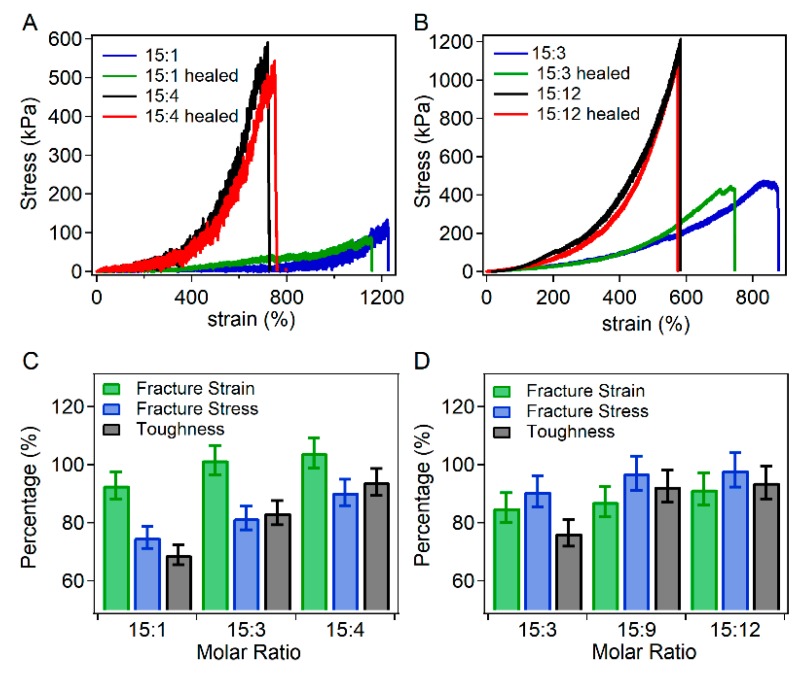
Self-healing properties of GGH and GHHPH hydrogels with different molar ratios of peptide and Zn^2+^ ions. (**A**) Stress–strain curves of GGH hydrogels at the strain rate of 30% min^−1^ before and after healing with different molar ratios of peptide and Zn^2+^ ions (15:1 and 15:4). The concentrations of GGH peptides and acrylamide were 50 mg mL^−1^ and 25 mg mL^−1^ respectively. (**B**) Stress–strain curves of GHHPH hydrogels at the strain rate of 30% min^−1^ before and after healing with different molar ratios of peptide and Zn^2+^ ions (15:3, 15:12). The concentrations of GGH peptides and acrylamide were 50 mg mL^−1^ and 25 mg mL^−1^, respectively. (**C**) Normalized recovery percentages of fracture strain, fracture stress and toughness with different ratios of GGH and Zn^2+^ ions. (**D**) Normalized recovery percentages of fracture strain, fracture stress and toughness with different ratios of GHHPH and Zn^2+^ ions.

Furthermore, the self-healing of hydrogels with different molar ratios of peptides and Zn^2+^ ions was also investigated in order to study the effects of Zn^2+^ ion concentrations on the self-healing properties (Figure 4). For GGH hydrogels, the molar ratios of peptides and Zn^2+^ ions were set as 15:1, 15:3 and 15:4, respectively. As shown in Figure 4C, the self-healing percentage of the fracture strain, fracture stress and toughness increased with the increasing molar ratios of Zn^2+^ ions and peptides, indicating that the self-healing ability was enhanced as the Zn^2+^ concentration increased. For GHHPH hydrogels, the molar ratios of peptide and Zn^2+^ ions were set as 15:3, 15:9, and 15:12 to ensure the ratios of histidine and Zn^2+^ ions were the same as those in GGH hydrogels (Figure 4B). Similarly, the self-healing percentages increased with the increase of Zn^2+^ concentrations (Figure 4D). All these results showed that the self-healing properties of the hydrogels can be further improved by the presence of additional metal ions, which increased the apparent association rates of peptides and Zn^2+^ ions.

## 4. Discussion

Although metal coordination bonds have been extensively used as reversible dynamic bonds for engineering self-healing hydrogels, a major challenge is that the resulting hydrogels have limited mechanical strength. However, the mechanical properties are essential for many biological applications, such as wound dressing and soft tissue medical adhesive, because the hydrogels are subjected to substantial mechanical stress in these applications. In this work, we showed that the toughness of the hydrogels can be greatly improved by using peptide cross-linkers of high coordination number (the number of binding sites in each cross-linking ligand). Due to the formation of multiple metal coordination bonds in each cross-linking ligand, the mechanical load can be evenly distributed to the bonds thus allowing them to bear larger overall mechanical forces. Therefore, the toughness of the hydrogels is significantly increased comparing to the single-bond cross-linkers. Moreover, due to the formation of specific metal ion binding sites, the association rates of peptides to metal ions are also faster than that of individual ligands. This leads to faster self-healing. Therefore, by simply tuning the coordination number (the number of binding sites in each cross-linking ligand), we were able to obtain hydrogels with combined high stretchability, toughness, and fast recovery. Note that this approach is distinct from the way of tuning the metal–ligand dynamics. It is worth noting that in our system, the maximum coordination number of Zn^2+^ ions is four. In order to form intermolecular cross-linking, the number of histidine residues in each peptide cannot be higher than four, which prevented us from testing hydrogels cross-linked ligands with other coordination numbers. To further validate this conclusion, future work using synthetic ligands and metal ions with higher coordination numbers is required.

Although it is possible to increase the toughness of hydrogels by making the metal coordination bonds less dynamic, the self-healing properties are compromised because the reassociation of the metal coordination bonds is retarded. Therefore, our approach provides a novel and efficient means for the construction of tough yet self-healable hydrogels. We envision that there is much room to tune the mechanical properties of the self-healing hydrogels by optimizing the cooperativity of dynamical interactions at the molecular level. For example, using nanoparticle cross-linkers or multivalent protein domains as the cross-linkers may further boost the mechanical features of self-healing hydrogels, which are certainly our next endeavors.

## 5. Conclusions

In summary, we have engineered highly stretchable, tough and self-healing hydrogels based on the coordination of peptides and metal ions. We showed that by increasing the coordination number of the binding sites (the number of binding sites in each cross-linking ligand), the toughness of the hydrogels can be greatly improved without sacrificing the stretchability and self-healing of the hydrogels. The resulting hydrogels can achieve a rigorous set mechanical characteristic, including the Young’s modulus of 15 kPa, the fracture strain of 620%, the toughness of 1350 kJ m^−3^, and the self-healing time of ~1 h. These properties may allow the hydrogels to be used as artificial skins or bioactive coatings for wound dressing. Moreover, the mechanical and self-healing properties can be tuned by adjusting the concentrations of the peptides and metal ions to match the mechanical properties of other biological tissues and to explore other biomedical applications. The concept of using higher number of binding sites in each cross-linking ligand to enhance the mechanical strength of self-healing hydrogels may open doors to design and engineer mechanically tough, highly stretchable and fast self-healing hydrogels.

## Figures and Tables

**Table 1:** Degree of polymerization, solid content, and mechanical properties of GGH and GHHPH hydrogels of different peptide concentrations.

Mass Concentration of Peptide (mg mL^−1^)		Polymerization Degree (%)	Solid Content (%)	Tension Strain Limit (%)	Tension Stress Limit (kPa)	Young’s Modulus (kPa)	Toughness (kJ m^−3^)
**GGH**	25	32.8 (M_n_ = 21,156)	25.84 ± 2.29	899.8 ± 56.1	18.6 ± 1.61	1.1 ± 2.9	90.6 ± 8.9
	50	36.2 (M_n_ = 17,521)	31.69 ± 2.56	793.5 ± 63.4	282.9 ± 37.6	6.1 ± 3.6	868.1 ± 23.3
	75	42.7 (M_n_ = 18,318)	31.39 ± 2.13	687.5 ± 54.9	220.3 ± 22.1	18.2 ± 4.2	586.7 ± 15.1
**GHHPH**	25	23.9 (M_n_ = 28,522)	22.79 ± 2.05	781.0 ±70.5	208.8 ± 23.7	7.1 ± 3.5	630.6 ± 50.4
	50	31.2 (M_n_ = 27,930)	26.67 ± 1.89	619.1 ±49.5	561.2 ± 44.9	14.9 ± 7.1	1348.4 ± 107.8
	75	27.4 (M_n_ = 21,804)	25.62 ± 2.03	434.1 ± 34.5	479.5 ± 38.4	122.9 ± 15.1	855.2 ± 82.1

## Supplementary Materials

The Supplementary Materials are available online at https://www.mdpi.com/2313-7673/4/2/36/s1: Figure S1. Dynamic mechanical properties of GGH and GHHPH Pre-gels without Zn^2+^ ion; Figure S2. SEM images of lyophilized GGH and GHHPH hydrogels; Figure S3. Rheological mechanical properties of GGH and GHHPH hydrogels in frequency sweep experiments; Figure S4. Rheological mechanical properties of GGH and GHHPH hydrogels in strain sweep experiments; Figure S5. Rheological mechanical properties in frequency sweep experiments, tensile experiments and self-healing properties of the covalent hydrogels; Figure S6. Rheological mechanical properties of GGH and GHHPH hydrogels in destroy–recovery experiments; Figure S7. Tensile experiments with multiple cyclic loading; Table S1. Details of all parameters and formulations in the article.
