# Supplementary material for: A Highly Stretchable, Tough, Fast Self-Healing Hydrogel Based on Peptide–Metal Ion Coordination
Source: Biomimetics (Basel). 2019 May 10;4(2):36. doi: 10.3390/biomimetics4020036 (PMC6632049; doi:10.3390/biomimetics4020036)
Supplement: Supplementary file 1 [file biomimetics-04-00036-s001.pdf]

Supporting information

A Highly stretchable, tough and fast self-healing hydrogel based on peptide-metal ion coordination

Liang Zeng, Mingming Song, Jie Gu, Zhengyu Xu, Bin Xue, Ying Li and Yi Cao

Supporting Figures

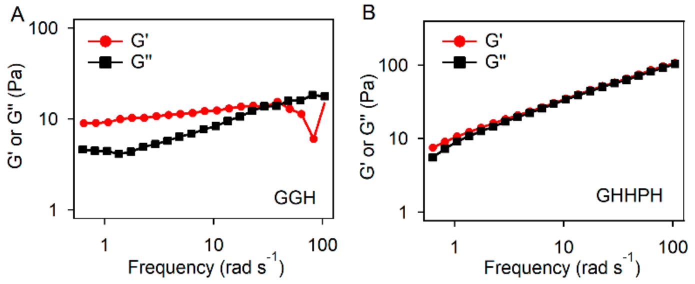

Figure S1. Dynamic mechanical properties of GGH and GHHPH Pre-gels without Zn^{2+} ion. (A) G′ and G″ of the GGH Pre-gels without Zn^{2+} measured in frequency sweep experiments (from 0.01 to 100 rad s^{-1}, 0.1% strain) at the peptide concentration of 50 mg mL^{-1} and the acrylamide concentration of 25 mg mL^{-1}. (B) G′ and G″ of the GHHPH Pre-gels without Zn^{2+} measured in frequency sweep experiments (from 0.01 to 100 rad s^{-1}, 0.1% strain) at the peptide concentration of 50 mg mL^{-1} and the acrylamide concentration of 25 mg mL^{-1}.

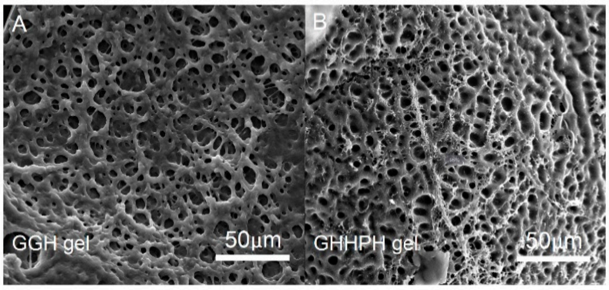

Figure S2. SEM images of lyophilized GGH and GHHPH hydrogels. (A) SEM images of lyophilized GGH hydrogels. (B) SEM images of lyophilized GHHPH hydrogels.

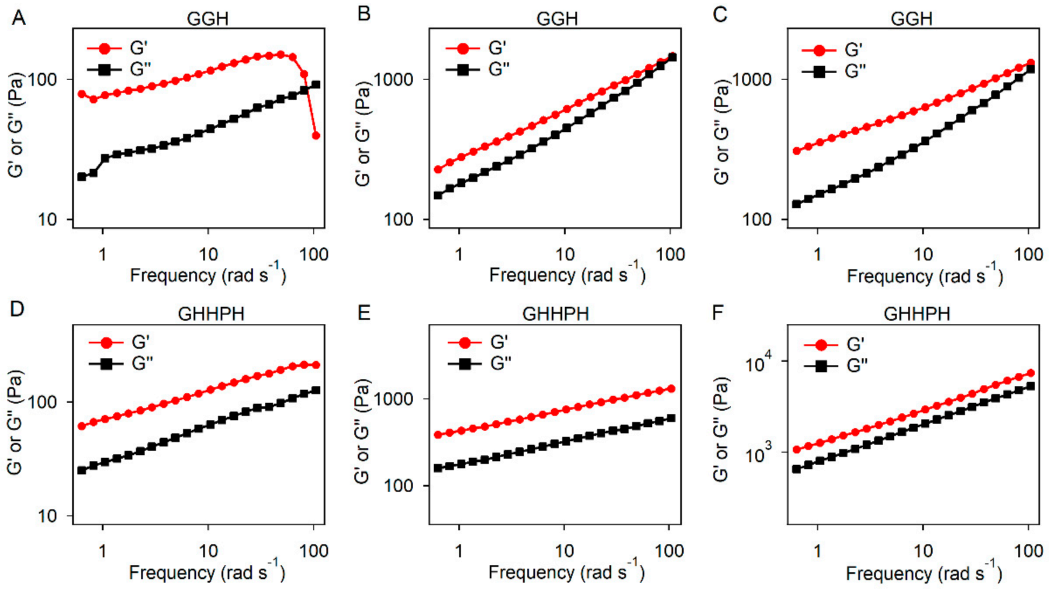

Figure S3. Rheological mechanical properties of GGH/GHHPH hydrogels at different concentrations in frequency sweep experiments. (A-C) G′ and G″ of GGH hydrogels measured in frequency sweep experiments (from 0.01 to 100 rad s^{-1}, 0.1% strain) at peptide concentrations of 25, 50 and 75 mg mL^{-1} respectively while the concentration of acrylamide was always 25 mg mL^{-1} and the molar ratio of peptides and ZnCl2 was always 15:3. (D-F) G′ and G″ of the hydrogels measured in frequency sweep experiment (from 0.01 to 100 rad s^{-1}, 0.1% strain) at peptide concentrations of 25, 50 and 75 mg mL^{-1} respectively while the concentration of acrylamide was always 25 mg mL^{-1} and the molar ratio of peptides and ZnCl2 was always 15:9.

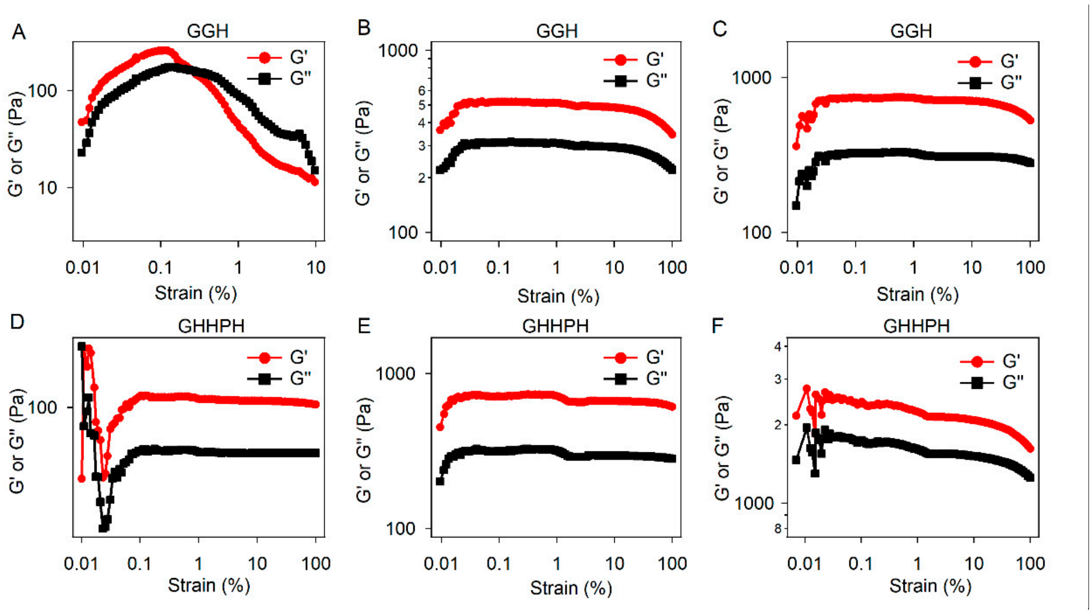

Figure S4. Rheological mechanical properties of GGH/GHHPH hydrogels at different concentrations in strain sweep experiments. (A–C) G′ and G″ of GGH hydrogels measured in a strain sweep experiment (from 0.01 to 100 %, 6.28 rad s^{-1}) at the peptide concentrations of 25, 50 and 75 mg mL^{-1} respectively while the concentration of acrylamide was always 25 mg mL^{-1} and the molar ratio of peptides and ZnCl2 was always 15:3. (D–F) G′ and G″ of GHHPH hydrogels measured in a strain sweep experiment (from 0.01 to 100 %, 6.28 rad s^{-1}) at the peptide concentrations of 25, 50 and 75 mg mL^{-1} respectively while the concentration of acrylamide was always 25 mg mL^{-1} and the molar ratio of peptides and ZnCl2 was always 15:9.

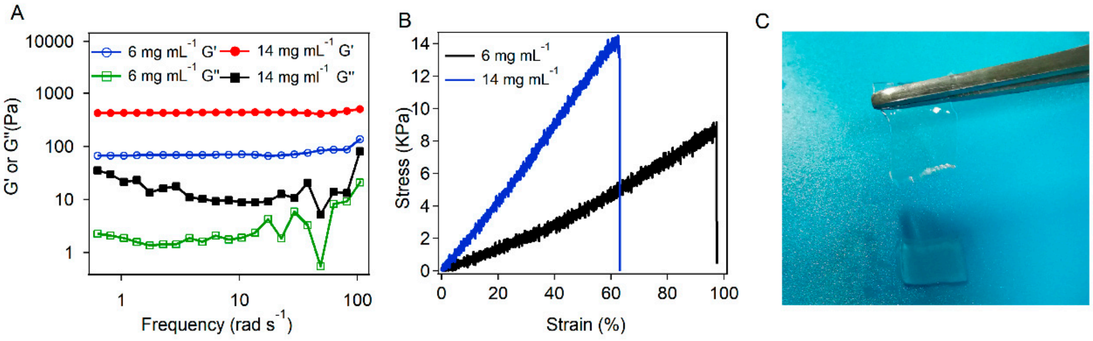

Figure S5. Rheological mechanical properties in frequency sweep experiments, and tensile experiments and self-healing properties of the covalent hydrogels. (A) G′ and G″ of the covalent hydrogels measured in a frequency sweep experiment (from 0.01 to 100 rad s^{-1}, 0.1% strain) at the bis-acrylamide concentration of 6 and 14 mg mL^{-1} while the concentration of acrylamide was always 25 mg mL^{-1}. (B) Stress-strain curves of covalent hydrogels at the bis-acrylamide concentration of 6 and 14 mg mL^{-1} while the concentration of acrylamide was always 25 mg mL^{-1}. (C) The optical image of a covalently cross-linked hydrogel that cannot self-heal after cutting.

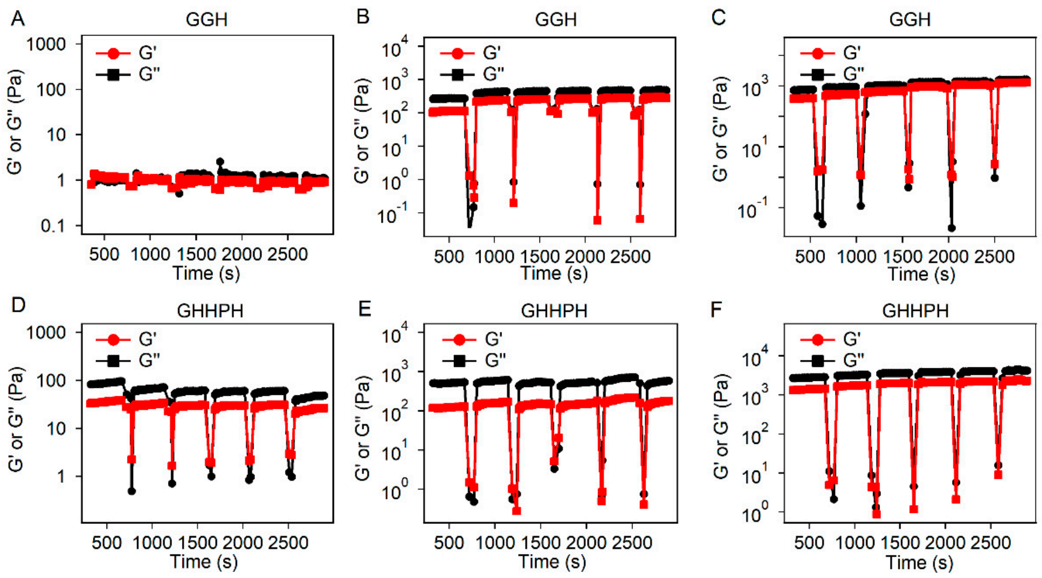

Figure S6. Rheological mechanical properties of GGH/GHHPH hydrogels at different concentrations in destroy-recovery experiments. (A–C) G′ and G″ of GGH hydrogels measured in a destroy-recovery experiment at the peptide concentration of 25, 50 and 75 mg mL^{-1} respectively while the concentration of acrylamide was always 25 mg mL^{-1} and molar ratio of peptides and ZnCl2 was always 15:3. The strain was set to an amplitude of 1000% to destroy the hydrogels for 60 seconds and switched back to an amplitude of 0.1% to monitor recovery of the mechanical properties for 300 seconds. The G′ and G′′ were measured at the frequency of 6.28 rad s^{-1} and the strain of 0.1% at 20 °C. (D–F) G′ and G″ of GHHPH hydrogels measured in a destroy-recovery experiment at the peptide concentration of 25, 50 and 75 mg mL^{-1} respectively while the concentration of acrylamide was always 25 mg mL^{-1} and molar ratio of peptides and ZnCl2 was always 15:9. The strain was set to an amplitude of 1000% to destroy the hydrogels for 60 seconds and switched back to an amplitude of 0.1% to monitor recovery of the mechanical properties for 300 seconds. The G′ and G′′ were measured at a frequency of 6.28 rad s^{-1} and the strain of 0.1% at 20 °C.

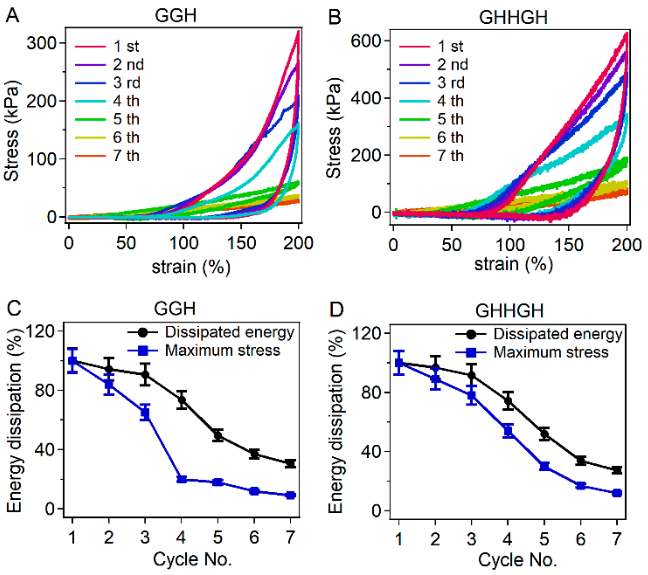

Figure S7. Tensile experiments with multiple cyclic loading. (A) Consecutive uniaxial stretching-relaxation cycles of GGH gels without any waiting time at the strain rate of 30% min^{-1}. The concentrations of GGH peptides and acrylamide were 50 mg mL^{-1} and 25 mg mL^{-1} respectively while the molar ratio of peptides and ZnCl2 was always 15:3. (B) Consecutive uniaxial stretching-relaxation cycles of GHHGH gels without any waiting time at the strain rate of 30% min^{-1}. Consecutive uniaxial stretching-relaxation cycles of GGH gels without any waiting time at the strain rate of 30% min^{-1}. The concentrations of GHHPH peptide and acrylamide were 50 mg mL^{-1} and 25 mg mL^{-1} respectively while the molar ratio of peptides and ZnCl2 was always 15:9. (C) The normalized dissipated energy and maximum stress of GGH hydrogel during the multiple cyclic loading. The concentrations of GGH peptide and acrylamide were 50 mg mL^{-1} and 25 mg mL^{-1} respectively while the molar ratio of peptides and ZnCl2 was always 15:3. (D) The normalized dissipated energy and maximum stress of GHHGH hydrogel during the multiple cyclic loading. The concentrations of GHHPH peptides and acrylamide were 50 mg mL^{-1} and 25 mg mL^{-1} respectively while the molar ratio of peptides and ZnCl2 was always 15:9.

Table S1. Details of all parameters and formulations in the article.

Parameter	Definition	Formulation
$\boldsymbol{L_0}$	The original length of hydrogel in the tension	N/A*
$\boldsymbol{L_b}$	The length of hydrogel at the break point	N/A
$\boldsymbol{x_0}$	The starting point of tension	N/A
$\boldsymbol{x_f}$	The fracture point of the tension	N/A
$\boldsymbol{Load}$	The force applied to the specimen	N/A
$\boldsymbol{s}$	The cross-sectional area of the specimen	N/A
$\boldsymbol{\sigma}$	The stress during the tension	N/A
$\boldsymbol{\varepsilon}$	The strain during the tension	N/A
$\boldsymbol{W_0}$	The weight of wet hydrogel	N/A
$\boldsymbol{W_d}$	The weight of dry hydrogel	N/A
$\boldsymbol{W_s}$	Solid content	$W_s = \frac{W_d}{W_0}$**
$\boldsymbol{\sigma}$	The stress during the tension	$\sigma = \frac{Load}{s}$***
$\boldsymbol{E_f}$	Toughness	$E_f = \int_{x_0}^{x_f} \sigma(x)dx$****

*N/A is not available.

** W_d is the weight of the lyophilized hydrogel and W_0 corresponds to the weight of the corresponding wet hydrogel.

*** *Load* corresponds to force applied to the specimen and s corresponds to the cross-sectional area of the specimen

****x_0 corresponds to the starting point of tension, x_f corresponds to the fracture point of the tension and σ corresponds to the stress during the tension.
